# Concurrent Mutations in *ATM* and Genes Associated with Common γ Chain Signaling in Peripheral T Cell Lymphoma

**DOI:** 10.1371/journal.pone.0141906

**Published:** 2015-11-04

**Authors:** Haley M. Simpson, Rashid Z. Khan, Chang Song, Deva Sharma, Kavitha Sadashivaiah, Aki Furusawa, Xinyue Liu, Sushma Nagaraj, Naomi Sengamalay, Lisa Sadzewicz, Luke J. Tallon, Qing C. Chen, Ferenc Livak, Aaron P. Rapoport, Amy Kimball, Arnob Banerjee

**Affiliations:** 1 Program in Oncology, Greenebaum Cancer Center, Department of Medicine, University of Maryland School of Medicine, Baltimore, MD, United States of America; 2 Center for Stem Cell Research and Regenerative Medicine, University of Maryland School of Medicine, Baltimore, MD, United States of America; 3 Genome Resource Center, Institute for Genome Sciences, University of Maryland School of Medicine, Baltimore, MD, United States of America; 4 Department of Pathology, University of Maryland School of Medicine, Baltimore, MD, United States of America; 5 Department of Microbiology and Immunology, University of Maryland School of Medicine, Baltimore, MD, United States of America; Mayo Clinic, UNITED STATES

## Abstract

Peripheral T cell lymphoma (PTCL) is a heterogeneous malignancy with poor response to current therapeutic strategies and incompletely characterized genetics. We conducted whole exome sequencing of matched PTCL and non-malignant samples from 12 patients, spanning 8 subtypes, to identify potential oncogenic mutations in PTCL. Analysis of the mutations identified using computational algorithms, CHASM, PolyPhen2, PROVEAN, and MutationAssessor to predict the impact of these mutations on protein function and PTCL tumorigenesis, revealed 104 somatic mutations that were selected as high impact by all four algorithms. Our analysis identified recurrent somatic missense or nonsense mutations in 70 genes, 9 of which contained mutations predicted significant by all 4 algorithms: *ATM*, *RUNX1T1*, *WDR17*, *NTRK3*, *TP53*, *TRMT12*, *CACNA2D1*, *INTS8*, and *KCNH8*. We observed somatic mutations in *ATM* (ataxia telangiectasia-mutated) in 5 out of the 12 samples and mutations in the common gamma chain (γ_c_) signaling pathway (*JAK3*, *IL2RG*, *STAT5B*) in 3 samples, all of which also harbored mutations in *ATM*. Our findings contribute insights into the genetics of PTCL and suggest a relationship between γ_c_ signaling and ATM in T cell malignancy.

## Introduction

Peripheral T cell Lymphoma (PTCL) accounts for 10–15% of Non-Hodgkin’s Lymphoma with about 7,000 cases diagnosed per year in the United States [1]. With at least 20 different subtypes currently recognized under WHO classification updated in 2008, PTCL represents a heterogeneous group of mature T and NK cell neoplasms with overall poor prognoses [2]. Traditionally, PTCL has been treated similarly to B cell lymphomas with various CHOP (cyclophosphamide, doxorubicin, vincristine, prednisone) based chemotherapy regimens and no targeted therapeutics effective against more than a subset of cases are currently available. While there is variability in outcome based on subtype, reported 5-year overall survival (OS) remains <40% [2, 3]. Recent clinical trials of alternative combinations of cytotoxic chemotherapeutics and newer therapeutic approaches tested thus far, including monoclonal antibodies such as anti-CD52, anti-CD30, and anti-CD4; immunoconjugates such as denileukin diftitox and brentuximab vedotin; epigenetic modifiers including HDAC inhibitors; signaling inhibitors such as Syc and protein kinase C inhibitors; and immunosuppressive (cyclosporine) and immunomodulatory agents (lenalidomide) are promising but have not yet led to substantial improvements in OS for PTCL patients [1, 2, 4]. Thus, there is a critical need for insights from the genomics of PTCL to facilitate the discovery of novel, personalized therapeutic targets and approaches.

To identify oncogenic mutations, and by extension, therapeutic targets in PTCL, recent studies have employed single gene, whole exome, or genome wide sequencing techniques in cases of PTCL [5–16]. These studies have identified mutations in a wide variety of genes including *STAT3*, *STAT5B*, *JAK1*, *JAK3*, *FYN*, *RHOA*, *NOTCH1*, *CD58*, *B2M*, *PLCG1*, *PTPN2*, *EZH2*, *FBXW10*, *TET2*, *DNMT3A*, *IDH2*, *ATM*, *CHEK2*, and p53 related genes (*TP53*, *TP63*, *CDKN2A*, *WWOX*, *and ANKRD11)* in different subtypes of PTCL. Due to the relatively low incidence of PTCL, however, the discovery cohorts within these studies are limited, with relatively small numbers of primary PTCL samples subjected to high throughput sequencing. Given the limited number of PTCL samples sequenced relative to many other cancers, further sequencing studies serve both to validate identified driver mutations and to discover novel mutations. Therefore, it is critical to compare and analyze mutations identified across independent studies to help understand the complete role of oncogenic mutations in PTCL. We conducted whole exome sequencing of 12 PTCL cases from untreated patients, compared to patient-derived non-tumor control cells, to identify somatic mutations: potential oncogenic drivers of PTCL.

## Materials and Methods

### Primary PTCL specimens

Specimens were collected for this study from patients diagnosed with PTCL at the University of Maryland Greenbaum Cancer Center with the approval of the University of Maryland, Baltimore Institutional Review Board (UMB IRB). Written consent was obtained from all patients involved in the study using a consent procedure approved by the UMB IRB. Documentation of the consent process includes patient, patient study number (samples are de-identified prior to use), principal investigator/designee signature, and date. Pathological samples used for analysis include patient blood, bone marrow, or lymph node tissue (S1 Table). Mononuclear cells were isolated from each specimen by subjecting single cell suspensions to Ficoll gradient centrifugation.

### Flow cytometry and cell sorting

Cells were stained with fluorophore-labeled antibodies to cell surface molecules for separation of malignant PTCL and non-malignant cell populations (B cell, monocyte) by flow cytometry and cell sorting. Surface antigens used to distinguish PTCL cells and non-malignant cells included CD2, CD3, CD4, CD5, CD7, CD8, CD14, CD19, CD30, CD45, and CD52 (S1 Table). All fluorophore-labeled antibodies were purchased from eBioscience. Cell sorting was performed using two-laser FACSAria I or three-laser FACSAria II cell sorters.

### Genomic DNA extraction

Cells were washed and resuspended in PBS. After addition of Proteinase K and RNAse A (Qiagen), genomic DNA was isolated using a DNeasy kit (Qiagen) per manufacturers’ instructions.

### Exome sequencing

Sequencing library construction, exome capture, sequencing, and analyses were carried out by the Genomics Resource Center (GRC) within the Institute for Genome Sciences (IGS) at the University of Maryland School of Medicine. Genomic DNA libraries with 7bp molecular barcode indexes were constructed for sequencing on the Illumina platform using the NEBNext^®^ DNA Sample Prep Master Mix Set 1 (New England Biolabs, Ipswich, MA). DNA was fragmented with the Covaris E210 focused ultrasonicator (Covaris Woburn, MA), targeting a size of 200bp, and libraries were prepared using a modified version of manufacturer’s protocol.

Following library construction, targeted capture was performed with the Agilent SureSelect Human All Exon V4 kit following the manufacturer’s protocol. Libraries were pooled so that each received ¼ or ½ a lane of sequencing, and were sequenced with an Illumina HiSeq2000 sequencer 100PE run, generating an average of 86.8 million passed-filter reads per sample.

Raw data from the sequencer was processed using Illumina’s RTA and CASAVA pipeline software and reads were truncated where the median quality score fell below Q20. Initial alignment to the hg19 human reference genome (GRch37) using BWA (v0.5.9) was followed by GATK (v1.4.5) for indel realignment and base quality score recalibration and Picard MarkDuplicates to remove artificially duplicated library fragments caused by PCR. The average on-target coverage for all samples was 94.4x and >87% of targets were covered at ≥20x with fewer than 4% of targeted bases lacking coverage. Somatic variants were predicted using both VarScan (v.2.3.7) and MuTect (v1.1.7). The resulting variant sets were annotated using ANNOVAR (v 2014-11-12). On average, VarScan found 208 somatic variants per sample, while MuTect found 264.

### Coverage and filtering for calling algorithms

MuTect uses a coverage cutoff of at least 14 reads in the tumor sample and at least 18 reads in the non-malignant cell sample and pre-applies filters to eliminate false positives. Only the high confidence set of calls that did not fail any of the MuTect filters were included in analysis.

VarScan uses a minimum coverage of 6 reads in the tumor sample and 8 reads in the non-malignant sample. We then applied the processSomatic tool to extract a high confidence set of variants. A somatic variant was considered high confidence if the variant allele frequency of at least 10% in tumor (default) and Fisher’s Exact Test P-value was < 0.07 (default) [17]. In addition, a threshold was applied for maximum variant allele frequency in the non-malignant samples, determined by the assessed purity of these samples. A false positive filter was then applied to the high confidence call set to remove any false positive variant calls due to sequencing or alignment related artifacts [17].

All variants called by Mutect and/or VarScan were then filtered by population frequency using the 1000 Genomes Project database to exclude variants with allele frequency > 0.01 in the population [18]. Application of this filter excluded 1% of the calls by Mutect and 18% of the calls by VarScan. A total of 3,137 calls by Mutect and 2,054 calls by Varscan (including mutations identified by both calling algorithms) remained for our analysis.

### Algorithms to predict potential cancer driver mutations and impact on protein function

Non-synonymous mutations identified were formatted for analysis according to the websites’ instructions and then queried by each of the following computational algorithms: Polyphen2 (Polymorphism Phenotyping v2), PROVEAN (Protein Variation Effect Analyzer), MutationAssessor, and CHASM (Cancer-Specific High-throughput Annotation of Somatic Mutations), accessed at http://genetics.bwh.harvard.edu/pph2/, http://provean.jcvi.org/index.php, http://mutationassessor.org/, and http://www.cravat.us/, respectively. Indels were only analyzed by PROVEAN, as the other algorithms are equipped only for missense mutation analysis.

## Results

### Whole exome sequencing of PTCL

To identify potential oncogenic mutations in PTCL, we performed whole exome sequencing of matched tumor and non-malignant DNA samples from 12 untreated patients with PTCL. Eight different PTCL subtypes were represented in our patient cohort, including one patient each with hepatosplenic T cell lymphoma (HSTL), T-cell large granular lymphocytic leukemia (T-LGL), lymphoepithelioid T cell lymphoma (LETL), Alk(+) anaplastic large cell lymphoma (ALCL), adult T-cell leukemia/lymphoma (ATLL), and Sezary Syndrome (SS), and three patients each with T-cell prolymphocytic leukemia (T-PLL) and peripheral T-cell lymphoma not otherwise specified (PTCLnos).

**Fig 1:**
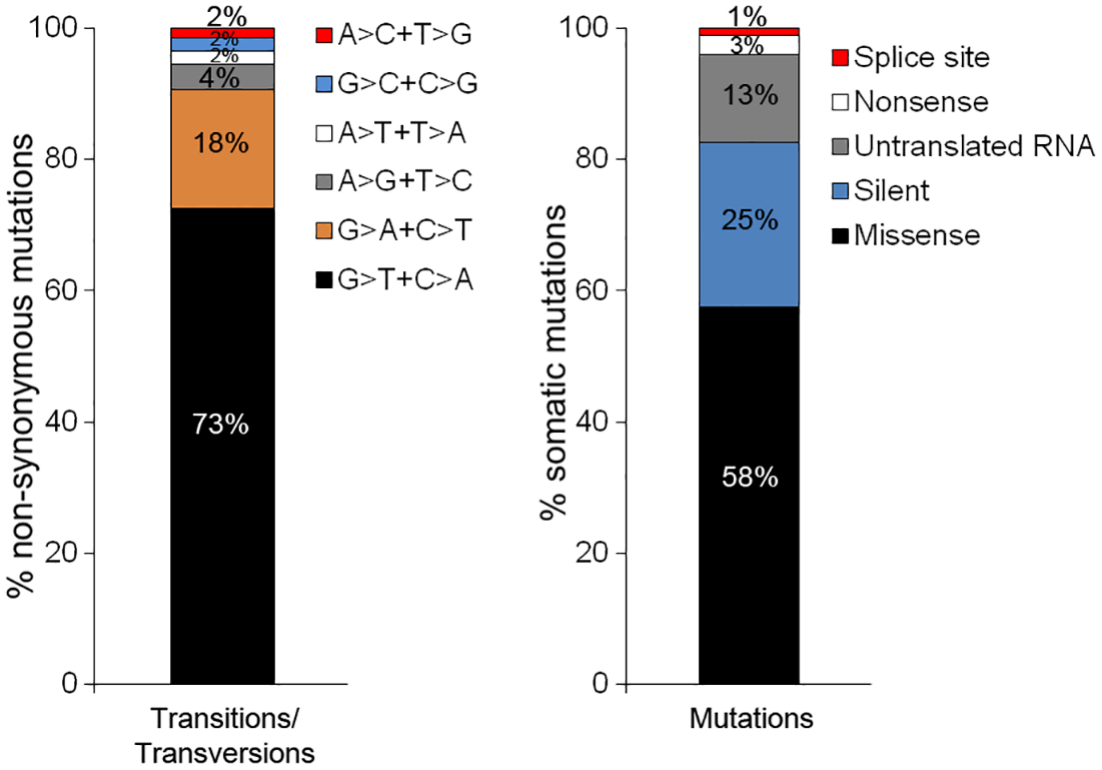
Characterization of somatic mutations in PTCL. (A) Bar graph shows percent of specified transitions and transversions resulting in non-synonymous somatic mutations identified by Mutect and VarScan in PTCL samples. (B) Bar graph shows percentage of each type of somatic mutation identified in PTCL samples.

Using two different calling algorithms, Mutect and VarScan, we detected a total of 1,245 unique, high-confidence, non-synonymous SNVs and 59 indels that passed our filters, across the 12 PTCL cases with an average of 93 non-synonymous somatic mutations per PTCL sample (range 15–340). More non-synonymous mutations were independently identified by Mutect (1,110) than VarScan (499); 333 mutations were identified by both calling algorithms. The most common transition/transversion resulting in non-synonymous exonic mutation was G>T+C>A (Fig 1A). The significance of this mutational pattern is not fully understood and other sequencing studies suggest that this transition/transversion is less than half as common as G>A+C>T in a wide variety of cancers [19, 20]. Approximately 71% of the mutations identified by Mutect and VarScan, are missense and nonsense mutations (Fig 1B). Of other mutations identified by the calling algorithms 25% are silent mutations, 1% are splice site mutations, and 13% are mutations in non-translated RNA. In subsequent analysis, we focused on the missense and nonsense mutations in protein coding regions due to their increased likelihood to result in functional protein changes. Silent, non-polymorphic mutations, however, may still affect transcription, translation, mRNA transport, or splicing and if the variant results in the need for a rare tRNA it may delay translation enough to cause the variations in protein folding [21]. We include a list of all recurrent genes with synonymous mutations by subtype that were identified in our patient cohort (S2 Table).

### Prediction of functionally important variants in PTCL

**Fig 2:**
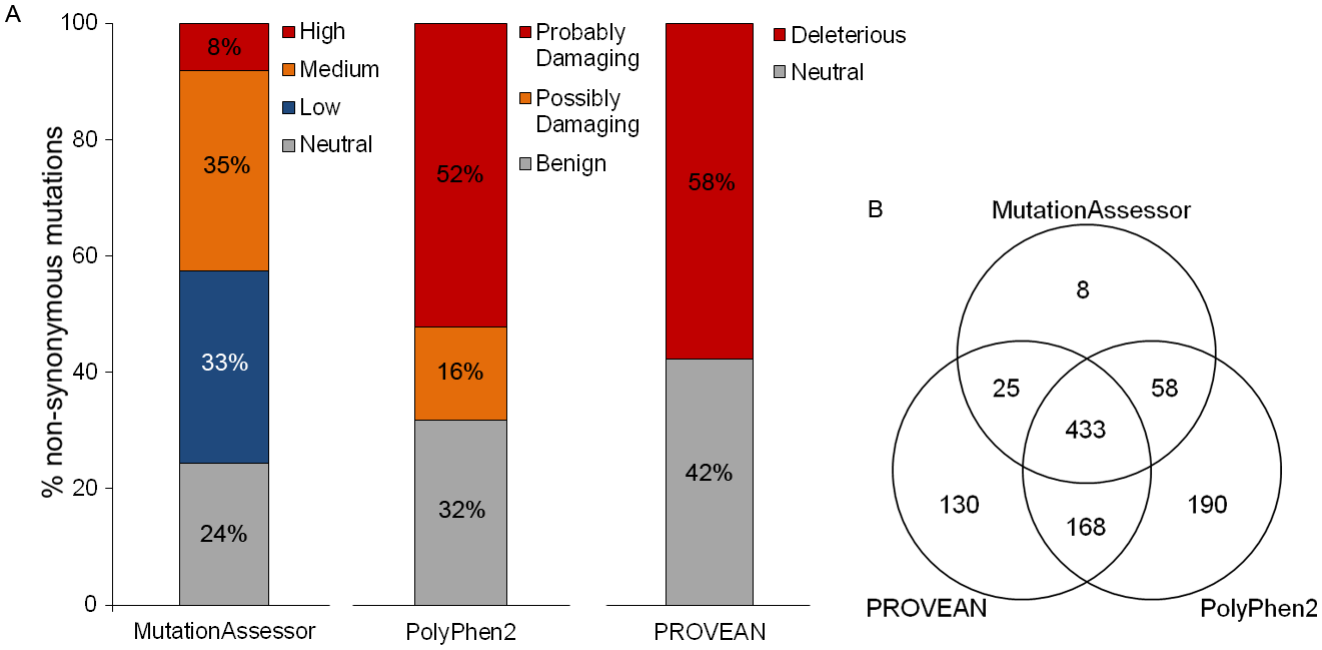
Functional algorithms, MutationAssessor, PolyPhen2, and PROVEAN, predict the majority of somatic mutations identified to significantly impact protein function. (A) Bar graph shows percent of non-synonymous somatic mutations and their probability to impact protein function, as predicted by MutationAssessor, PolyPhen2, and PROVEAN. (B) Venn diagram shows number of non-synonymous somatic mutations predicted to significantly impact protein function by each algorithm or combination of algorithms: MutationAssessor, PolyPhen2, and PROVEAN. Mutations were considered significant if selected as “high” or “medium”, “probably damaging” or “possibly damaging,” and “deleterious,” respectively.

To identify the non-synonymous SNVs from our samples most likely to influence protein function, we used three independent structure/homology-based algorithms: Polyphen2 PROVEAN, MutationAssessor, as well as a fourth algorithm, CHASM, designed to prioritize somatic missense mutations based on their representation in large-scale cancer sequencing studies [22–26]. CHASM uses COSMIC (Catalogue of Somatic Mutations in Cancer) as a training set to predict whether a somatic missense mutation will contribute to the tumorigenicity of the malignant cell from which it was sequenced [24]. Of the 1,245 missense SNVs and 59 indels called, 154 (12%) were selected by CHASM as likely to be cancer driver mutations. PolyPhen2 predicts the functional impact of a missense mutation by comparison of 1) biochemical changes in protein structure between the wild-type and mutant allele based on predicted protein domains and 2) sequence homology based on evolutionary conservation of the wild-type allele between species [22]. Of the non-synonymous mutations identified, 52% were predicted to be “Probably Damaging” and 16% to be “Possibly Damaging” to protein function by PolyPhen2 (Fig 2A). MutationAssessor prioritizes relative mammalian evolutionary conservation over conservation between all species, to predict the probability of the mutation significantly impacting mammalian protein function [26]. This more conservative method predicted 8% of the mutations will have a “High” and 35% will have a “Medium” chance of significantly affecting protein functions (Fig 2A). PROVEAN compares homologous sequences, including the region surrounding substituted, added, or deleted amino acids, so that it may assess the potential for deleterious impact of SNVs and indels on protein function [23]. Of the SNVs and indels analyzed, 58% were predicted to be “Deleterious” using this algorithm (Fig 2A). Of the 1,245 SNVs, 433 SNVs were predicted by all three general algorithms, Polyphen2, PROVEAN, and MutationAssessor, to encode functionally relevant amino acid changes (Fig 2B). Of these, 104 were also selected by CHASM as likely to be cancer drivers (Table 1).

**Table 1:** 104 somatic mutations predicted to be cancer drivers that significantly alter protein function by all four algorithms.

Subtype	Mutation		Subtype	Mutation		Subtype	Mutation		Subtype	Mutation	
ALCL	ABCB1	Q475H	T-PLL	ATM	C2930F	T-PLL	MYO3A	P380H	T-PLL	TBC1D16	R76L
ALCL	EIF3M	G112W	T-PLL	ATM	R3008H^1^	T-PLL	NEK8	G86C	T-PLL	TBC1D22B	P303H
ALCL	RAB13	R79S	T-PLL	CA12	W62C	T-PLL	NMS	R69M	T-PLL	TESK2	G136W
ALCL	SLC22A4	G161W	T-PLL	CAMKMT	G255W	T-PLL	NT5C1B-RDH14	G353W	T-PLL	TNIK	K41N
ALCL	SUSD1	G67W	T-PLL	CAPN6	R454S	T-PLL	OSBP	P497Q	T-PLL	TNRC6C	W1487C
ALCL	VRK1	W261L	T-PLL	CNPY3	R95L	T-PLL	PCDHB3	R92L	T-PLL	TRIM2	P37H
ALCL	ZFHX4	R2659L	T-PLL	CNTNAP2	R157S	T-PLL	PFN2	R56L	T-PLL	TSTD2	P284Q
ATLL	ADSL	G54C	T-PLL	CREBBP	R1185S	T-PLL	PIM3	P213Q	T-PLL	UBP1	P142Q
ATLL	ALDOA	R60C	T-PLL	CUX1	R1259L	T-PLL	POPDC2	R216L	T-PLL	USP24	P124H
ATLL	DMBX1	R122C	T-PLL	DHX30	R918L	T-PLL	PRC1	P151T	T-PLL	USP4	P798Q
ATLL	KCNH4	R353L	T-PLL	DIAPH2	W643L	T-PLL	PRDM16	R173H	T-PLL	UTRN	R3348S
ATLL	MRPS33	R89S	T-PLL	DOCK7	G1343W	T-PLL	PREP	W150L	T-PLL	WDR17	W1265L
ATLL	NFATC3	W644C	T-PLL	ERLIN2	R169L	T-PLL	PREX2	W234L	T-PLL	ZBTB39	H556N
ATLL	PRKCB	D427N	T-PLL	FAM169A	G156W	T-PLL	PTPRD	G904V	PTCLnos	CACNA2D1	A248P
HSTL	C4orf27	G130W	T-PLL	FAM219A	R35L	T-PLL	RAB6B	G25W	PTCLnos	CLPX	G296V
HSTL	FKBP4	R359L	T-PLL	FBXL2	W42L	T-PLL	RAE1	W156L	PTCLnos	FBXW7	S464L
LETL	EGR2	H416D	T-PLL	FILIP1	D394G	T-PLL	RBL2	W205L	PTCLnos	FYN	G407R
LETL	NTRK3	P331L	T-PLL	HECW1	R1263L	T-PLL	RPN1	G325W	PTCLnos	HIF1A	R53L
SS	ESRRG	L67H	T-PLL	HTR3A	R245C	T-PLL	RUNX1T1	R349L	PTCLnos	INHBA	M418T
SS	PCDHGA5	E253K	T-PLL	ICA1	W242L	T-PLL	RUNX1T1	R229L	PTCLnos	KCNH8	Y671C
TLGL	CSNK2A1	R80H	T-PLL	INTS8	W548L	T-PLL	RXRB	R52L	PTCLnos	MAN1C1	P302S
TLGL	PGD	R112L	T-PLL	KCNJ6	R214L	T-PLL	SLC35C1	P73T	PTCLnos	MMP24	Y170C
TLGL	RTF1	M199K	T-PLL	KIT	W582L	T-PLL	SLC44A4	G525W	PTCLnos	PAPPA2	Q822K
TLGL	SCN2A	W386L	T-PLL	LIMK2	G520W	T-PLL	SPTLC3	R533L	PTCLnos	TBC1D22A	G306W
TLGL	SERPINA12	P76H	T-PLL	LZTS2	M26I	T-PLL	STRADB	P223Q	PTCLnos	TP53	V41M^2^
T-PLL	ATG13	W50L	T-PLL	MYO1B	R716L	T-PLL	TAF8	G73W	PTCLnos	TRMT12	P60L

CHASM significance determined by p≤0.05. If a mutation was previously identified, the appropriate citation is listed as a footnote [27–32].

^1^ Camacho, *et*. *al*. 2002; Navrkalova, *et*. *al*. 2013; Biankin, *et*. *al*. 2012; Fang, *et*. *al*. 2003; Gronbaek, *et*. *al*. 2002

^2^ Achatz, *et*. *al*. 2007

### Genes containing non-synonymous somatic mutations in multiple PTCL samples

**Fig 3:**
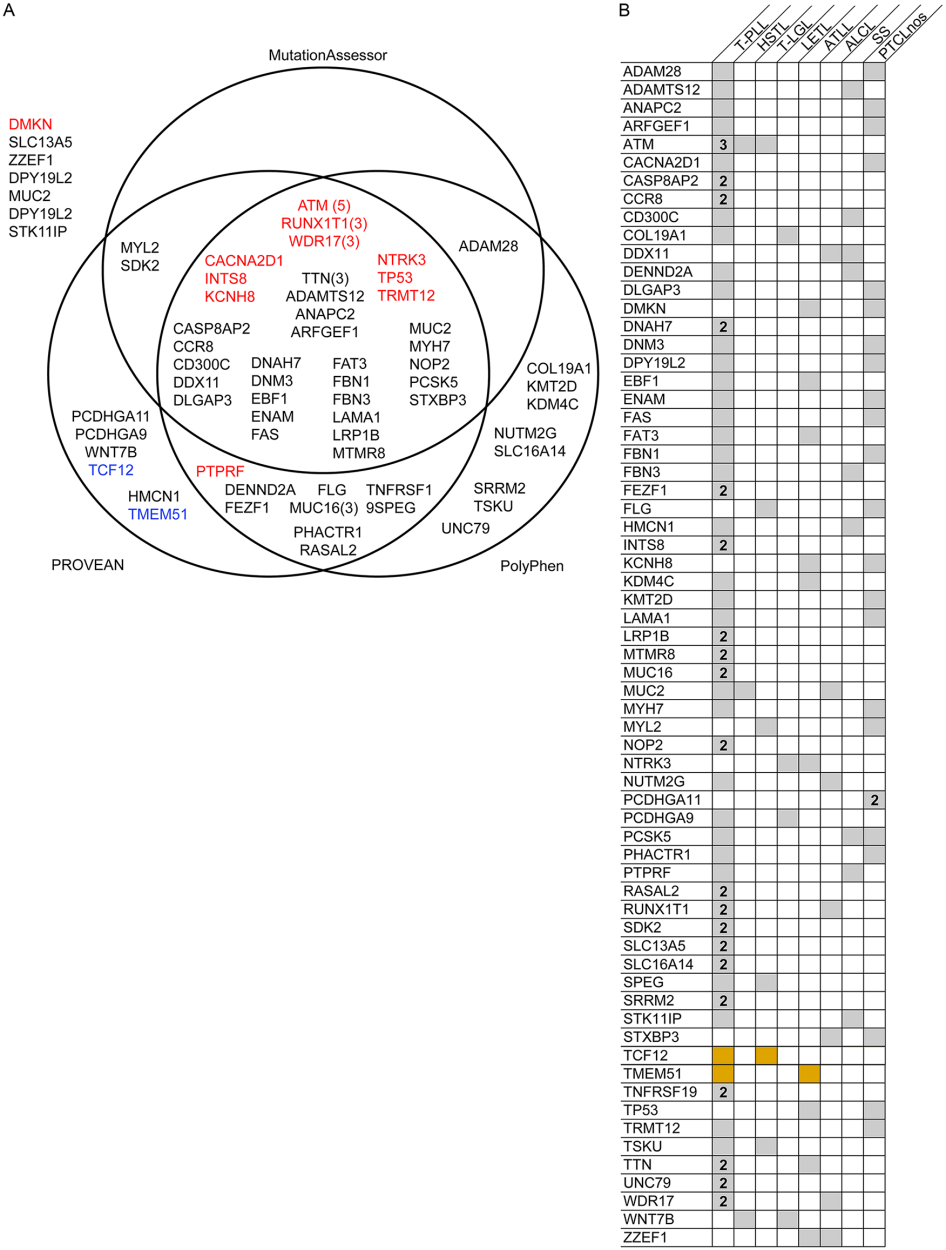
70 genes contain somatic mutations in more than one PTCL sample. (A) Venn diagram shows which algorithms predicted the mutations in genes somatically mutated in multiple PTCL cases to significantly alter protein function. Genes listed in red contain significant potential cancer driver mutations by CHASM (p≤0.05). Genes listed in blue contain identical SNVs in both samples. Genes were found to contain mutations in two cases, unless indicated otherwise with a number in parentheses. (B) Grid shows which PTCL subtypes (columns) contain the somatic mutations identified in genes mutated in more than one sample (rows) via grey shaded boxes. Gold shading indicates identical repeated SNVs identified in multiple cases/subtypes. Numbers in boxes indicate the number of samples of the indicated subtype containing a mutation in the indicated gene, if greater than one.

We identified 70 genes with missense or nonsense somatic mutations in more than one PTCL sample. For each of these genes, we determined whether the SNVs identified were predicted to have functional consequences by the Polyphen2, PROVEAN, MutationAssessor, or CHASM algorithms (Fig 3A). The most frequently mutated gene in our sample cohort is *ATM*, found to contain non-synonymous somatic mutations in 5 out of the 12 samples (42%). *ATM* has previously been shown to harbor somatic mutations in over 50% of sequenced tumor samples from patients with the T-PLL subtype of PTCL [12, 33–36]. Out of the 5 samples with *ATM* mutations in our data, three were from cases of T-PLL and the two others were from HSTL and T-LGL cases. *ATM* mutations in conserved residues were verified by Sanger sequencing (S1 Fig). Mutations in *RUNX1T1* (encoding cyclin-D-related protein, a transcriptional regulator) and *WDR17* (WD repeat-containing protein 17) were identified in 3 patient samples in the cohort and predicted to be likely cancer driver mutations impacting protein function by all 4 algorithms (Fig 3A). Mutations in *TTN* (titin, involved in chromosome segregation) were also identified in 3 samples and predicted to have a significant probability of impacting protein function by Polyphen2, PROVEAN, and MutationAssessor. Mutations in *MUC16* (mucin) were also observed in 3 samples and predicted to be significant by Polyphen2 and PROVEAN. Mutations in *CACNA2D* (a voltage-dependent calcium channel), *INTS8* (a component of small nuclear RNA transcription complex), *KCNH8* (a potassium voltage-gated channel), *NTRK3* (tyrosine-protein kinase receptor), *TP53* (p53), and *TRMT12* (a guanosine modifying transferase) were identified in 2 samples and predicted to be cancer driver mutations impacting protein function by all 4 algorithms (Fig 3A). Twenty of the 70 mutations identified were selected as a representative subset for Sanger sequencing validation, all of which were verified.

Our data contains three samples from patients with T-PLL, all with a mutation in *ATM* and two out of the three with mutations in *CASP8AP2* (CASP8-associated protein 2, TNFα signaling), *CCR8* (chemokine receptor), *DNAH7* (dynein heavy chain), *FEZF1* (fez family zinc finger protein), *INTS8*, *LRP1B* (receptor-mediated endocytosis), *MTMR8* (phosphatase), *MUC16*, *NOP2* (methyltransferase, cell cycle regulation), *RASAL2* (Ras GTPase-activating protein), *RUNX1T1*, *SDK2* (cell adhesion), *SLC13A5* (sodium/citrate cotransporter), *SLC16A14* (monocarboxylate transporter), *SRRM2* (pre-mRNA splicing), *TNFRSF19* (tumor necrosis factor receptor superfamily member), *TTN*, *UNC79* (sodium channel complex component), and *WDR17* (Fig 3B). Two of the 3 samples from patients with PTCLnos harbor mutations in *PCDHGA11* (a potential calcium-dependent cell-adhesion protein), but we did not identify any other recurrent mutations in this subtype.

Among the 70 genes found to contain somatic mutations in multiple samples, two genes contained identical somatic mutations in two different PTCL samples: TCF12 R300L (transcriptional regulator) and TMEM51 E169del (transmembrane protein) (Fig 3A, in blue; Fig 3B in gold). TCF12 R300L and TMEM51 E169del were predicted to significantly alter function of the protein by PROVEAN. These SNVs have not previously been identified.

### ATM mutations in PTCL

**Fig 4:**
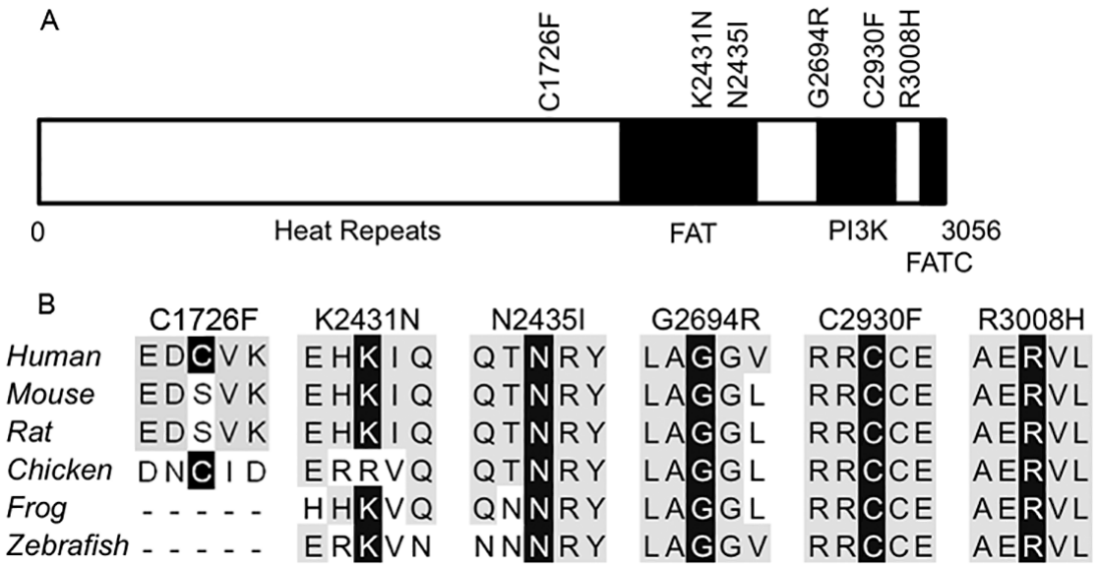
Somatic mutations in ATM, identified in PTCL patients, involve highly conserved residues. (A) Schematic representation of ATM protein domains showing location of somatic mutations in ATM from 5 different PTCL samples. (B) Multiple sequence alignment across species around the 6 mutations in ATM found in 5 samples from patients with PTCL. Conserved mutated residue highlighted in black, other conserved residues highlighted in grey.

*ATM* has been found to be mutated, or deleted, in the majority of cases of T-PLL, with most of the identified mutations clustering near the ATM PI3Kinase domain [12, 35]. Out of the five samples with *ATM* point mutations in our study, three are from patients with T-PLL, while the final two are from patients with HSTL and T-LGL. Three of the somatic mutations identified, two from T-PLL samples and one from the HSTL sample, are in or near the highly conserved kinase domain (Fig 4). The third T-PLL sample contains mutations in the FAT (FRAP-ATM-TRRAP) domain adjacent to the kinase domain. The T-LGL case contained a mutation upstream of the FAT domain, outside of the highly conserved region of the protein. Of the mutations, the R3008H mutation has been previously observed in multiple cancers, including pancreatic cancer, chronic lymphocytic leukemia, mantle cell lymphoma, and diffuse large B cell lymphoma and has been shown to decrease expression of ATM in mantle cell lymphoma, suggesting that it is associated with a loss-of-function [28–32]. Furthermore, the R3008 residue has been previously found to be mutated in cases of T-PLL, including a T-PLL case included in our analysis [37]. SNVs and indels within 10 bases of two SNVs we identified, N2435I and K2431N, have been associated with T-PLL as well as B-cell chronic lymphocytic leukemia [34, 38, 39]. In our data, loss-of-ATM-function is supported by the observation of homozygous mutation (loss of heterozygosity) at the *ATM* locus in the three T-PLL samples and all SNVs were predicted to significantly impact ATM protein function by at least one of the algorithms utilized.

As recurrent mutations in ATM and p53 related molecules have been identified in other studies of PTCL, we scanned our data for mutations in this pathway and identified mutations in several related genes: *TP53*, *BRAT1*, *CREBBP*, *MAPK9*, *MAPK14*, *NFKBIA*, and *TLK1*. p53 contained mutations in one case of PTCLnos and one of ATLL, encoding amino acid changes V41M and D10Y, respectively. p53 V41M was predicted to be a cancer driver mutation by CHASM and selected by all three general algorithms as likely to impact protein function.

### Common gamma chain (γ_c_) signaling pathway mutations in PTCL

**Fig 5:**
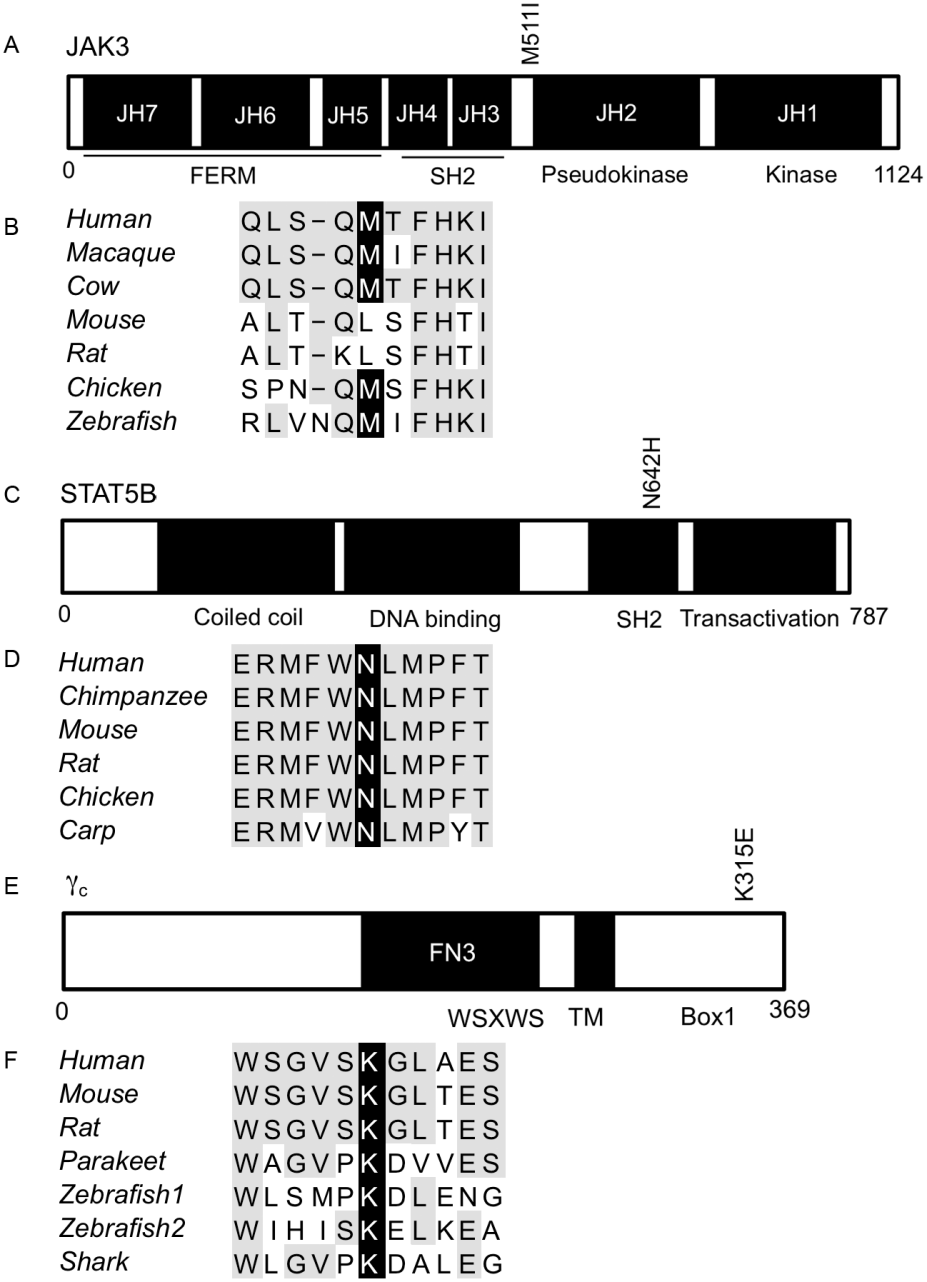
γ_c_ cytokine signal transducers contain mutations in highly conserved amino acid residues. (A) Schematic representation of JAK3 protein domains showing somatic mutation in identified in PTCL sample. (B) Sequence alignment of JAK3 M511 across 7 species. Conserved mutated residue highlighted in black, other conserved residues in grey. (C) Schematic representation of STAT5B protein domains showing somatic mutation identified in PTCL sample. (D) Sequence alignment of STAT5B N642 across 6 species. Conserved mutated residue highlighted in black, other conserved residues in grey. (E) Schematic representation of γ_c_ protein domains showing somatic mutation identified in PTCL sample. (F) Sequence alignment of γ_c_ K315 across 6 species. Conserved mutated residue highlighted in black, other conserved residues in grey.

JAK/STAT signaling pathway molecules (STAT3, STAT5B, JAK1, and JAK3) have previously been shown to be recurrently mutated in various subtypes of PTCL [5, 7–9, 12]. We observed mutations in three genes important in JAK/STAT signal transduction through the γ_c_ in our sample set: *IL2RG*, *JAK3*, and *STAT5B*, verified by Sanger sequencing (Fig 5 and S2 Fig).

In one of our samples from a patient with T-PLL we observed a somatic mutation in a conserved region of JAK3 adjacent to the pseudokinase domain, M511I (Fig 5A and 5B). JAK3 M511I has been previously described in T-PLL, SS, acute myeloid leukemia, and T-cell acute lymphoblastic leukemia (T-ALL), and has been characterized as an activating mutation, conferring cytokine independent growth to Ba/F3 cells and demonstrating transforming potential in murine hematopoietic progenitor cells [40–45]. CHASM predicted JAK3 M511I to be a cancer driver mutation (S3 Table).

In the HSTL sample in our analysis, we identified a somatic mutation in a highly conserved residue in the SH2 domain of STAT5B encoding a N642H mutation, a prolific oncogenic mutation found in many hematologic malignancies (Fig 5C and 5D). STAT5 N642H mutation was first identified as an activating mutation in an *in vitro* screen, then demonstrated to increase the transcriptional activity of STAT5A, which shares extensive sequence homology with STAT5B, and shown to allow cytokine independent growth of Ba/F3 cells [46]. STAT5B N642H has further been identified in multiple subtypes of PTCL: 2% of cases of T-LGL, 7/21 (33%) of cases of HSTL, and in T-PLL where it has demonstrated increased colony forming capacity in Jurkat cells, a T-cell leukemia cell line [8, 12, 47]. STAT5B N642H has also been found in 6.3% of cases of pediatric T-ALL, correlating with increased risk of relapse and decreased probability of event free survival [48]. Alternative studies do report variation in the rate of STAT5B N642H in pediatric T-ALL: from 1/64 (1.5%) to 1/4 (25%) [49, 50]. This mutation has also been identified in γδ-T-cell lymphomas where the mutant histidine has been shown to increase binding affinity for the activating phosphotyrosine, Y699, in the STAT5B molecule leading to persistence of mutant pSTAT5B and increased binding to targets [51]. STAT5B N642H mutation has also been observed in acquired aplastic anemia [52]. PolyPhen2 and PROVEAN predicted this mutation to have a “Probably Damaging” and “Deleterious” impact on the protein, respectively.

The γ_c_ K315E mutation we observed in a T-PLL case is in a highly conserved residue located in the intracellular region of the γ_c_ protein C-terminal to the box1 motif, which is required for JAK binding and activation (Fig 5E and 5F). This mutation was recently noted in one other case of T-PLL [12]. PolyPhen2 predicted γ_c_ K315E to be “Probably Damaging” and MutationAssessor indicated it has a “Medium” probability of having a significant impact on the protein’s function. All three of these γ_c_ signaling pathway mutations in *IL2RG*, *JAK3*, and *STAT5B* were found in patients who also had mutations in *ATM*. Possible significance of this finding is unknown.

## Discussion

Next generation and high throughput sequencing techniques are employed to gain insight into the biology of PTCL in an effort to develop a basis for novel therapeutic approaches. We performed whole exome sequencing of paired PTCL and non-malignant cell DNA from 12 untreated PTCL patients, representing a total of 8 different subtypes, in order to identify potential oncogenic mutations. Some of the mutations we identified in our study overlap with those identified in other current PTCL sequencing studies, lending validity to studies with relatively small sample sizes and supporting the potential importance of genes found to contain mutations in multiple studies in PTCL. Our results support and expand upon current understanding of the role of mutated genes in PTCL to help lay the groundwork for future development of targeted therapeutic strategies.

Somatic mutations in genes encoding molecules involved in cytokine signal transduction, and specifically in γ_c_ associated JAK-STAT signaling, have previously been identified in NK-/T-cell lymphoma (NKTCL), angioimmunoblastic T-cell lymphoma (AITL), T-PLL, HSTL, SS, and T-LGL subtypes of PTCL [5, 8, 12, 42, 47, 53]. We identified somatic mutations in JAK-STAT signaling molecules associated with γ_c_ signaling in T-PLL and HSTL. Other sequencing studies have revealed somatic activating mutations in JAK3 (A572V and A573V) in 38% of NKTCL, STAT3 and STAT5B in T-LGL, and recurrent gain-of-function mutations in JAK2 and STAT3 in AITL [5, 8, 42, 53]. The malignant transforming potential of JAK3 M511I has been shown to be dependent on the expression of the γ_c_ and to increase with γ_c_ overexpression [54]. Our study, taken together with recent work identifying the γ_c_ K315E mutation in T-PLL, as well as an indel in the γ_c_ that was demonstrated to increase STAT5 transcriptional activity, establishes a pattern of recurrent mutations in the γ_c_ in T-PLL that may drive the development of this malignancy [12]. As γ_c_ cytokine signaling, particularly in the context of IL-2, IL-7, and IL-15 signaling, is critical for T-cell differentiation, apoptosis, survival and proliferation, mutations altering this signal transduction pathway are well placed to potentially drive T cells to a cytokine-independent, malignant phenotype [55, 56]. Studies on PTCL are limited in scope by the relative rarity of the disease and the addition of our findings lends validity to these studies in the field where large validation sets are often unavailable. Our observation of activating mutations in JAK3 and STAT5B in T-PLL and HSTL, along with these prior studies, show that mutations leading to activation of γ_c_ associated JAK-STAT cytokine signaling pathways are present in at least 5 subtypes of PTCL and may represent a unifying trait in a disease marked by heterogeneity between subtypes.

Identification of gain-of-function mutations in the common gamma chain signaling pathway suggests utility of JAK/STAT pathway inhibitors in PTCL therapy. JAK inhibitors are currently in development and several are approved for patient use, such as ruxolitinib in the treatment of myelofibrosis and tofacitinib in rheumatoid arthritis [57, 58]. In light of the finding of activating mutations in JAK3 in multiple subtypes of PTCL, including our study, it will be important to investigate the use of these inhibitors for the treatment of these malignancies [42, 59]. Furthermore, STAT molecules have been shown to be constitutively activated in some hematologic malignancies, including STAT5 in several subtypes of PTCL, ~35% of cases of HSTL, 2% of T-LGL, and ~6% of pediatric T-ALL often leading to worse patient outcomes [8, 12, 47, 48]. As such, direct inhibition of STATs, such as with pimozide, may be an appealing therapeutic strategy as well [60]. Our results, in combination with the literature, support further investigation of the utility of JAK/STAT pathway inhibitors as a novel therapeutic intervention in PTCL.

The most frequently recurring mutated gene identified in our study is *ATM*, mutated in 5 out of 12 PTCL cases and predicted by algorithms as likely to impact protein function and drive oncogenesis. Another recent whole exome sequencing study of 12 PTCL samples also identified recurrent mutations in *ATM* [11]. One of the ATM mutations identified is R3008H, also found in our study, and included in the COSMIC database due to its known association with cancer. Cells with inactivating mutations in ATM can be selectively targeted with poly (ADP ribose) polymerase (PARP) inhibitor therapy [61]. PARP inhibitors, such as olaparib, are already in clinical use for treatment of solid tumors and in clinical trials for patients with chronic lymphocytic leukemia and T-PLL and could prove useful for treatment of many other malignancies driven by ATM deficiency. Furthermore, the three PTCL samples (two T-PLL, one HSTL) with mutations in γ_c_ signal transduction components in our study were among the samples containing mutations in *ATM*. Our study suggests that, due to overlap of mutations in ATM and the γ_c_ signaling pathway, there is a possible correlation between these two pathways in HSTL as well as T-PLL. In a study of HPV viral replication in hepatocytes, phosphorylated STAT5B was found to activate the ATM pathway through the action of peroxisome proliferator-activated receptor gamma (PPARγ) [62]. The mechanisms of the relationships between these pathways have not been explored in hematologic cancers or normal T-cell biology.

Our analysis has identified mutations in several of the highly recurrent mutated genes found in previous PTCL sequencing studies, supporting the importance of these pathways in PTCL oncogenesis. Aside from mutations in ATM, p53, and the γ_c_ signaling pathway, we also identified mutations in FYN kinase, NOTCH molecules, RHOA related molecules, and PLCG1, corroborating data published by several other studies [10, 11, 13, 14, 16]. The FYN G407R mutation we identified was predicted to be a cancer driver with a significant impact on protein function by all 4 algorithms. Although our data did not contain a mutation in NOTCH1 or RHOA, we did find NOTCH2, NOTCH4, RHOV and RHOBTB1 mutations, significant by PolyPhen2 and PROVEAN. We also identified a PLCG1 mutation, P658Q, likely to affect protein function by all 3 general algorithms. These findings are consistent with these other studies in the field, which are also relatively small scope, lending validity to our analysis. Because many PTCL studies are limited by the rarity of the disease, inter-study comparison can help address issues of interpretation of a small study when validation sets are unavailable and demonstrate novel, related somatic mutations in PTCL that may be drivers of PTCL oncogenesis.

We also identified recurrent mutations in *MUC16* and *TTN*. However, these genes have been found to be recurrently mutated in many different whole exome sequencing studies as they are quite long (22,152 and 34,350 amino acids, respectively) and therefore have an increased tendency to accumulate more variants [63]. Thus, despite being selected by the algorithms, we consider these mutations unlikely to be cancer drivers of PTCL.

We used four algorithms, PROVEAN, MutationAssessor, PolyPhen2, and CHASM, as a tool to predict mutations that have a higher chance of driving PTCL oncogenesis [22–26]. Our analysis suggests that while algorithms may be useful in identifying mutations with a significant impact on protein function and tumorigenesis, mutations should not necessarily be dismissed from consideration as cancer drivers if not selected by these algorithms. It is possible that these computational programs are not as powerful in the identification of activating mutations, as JAK3 M511I and STAT5B N642H mutations were each predicted to be significant by only one of the algorithms, even though they have been previously characterized as activating mutations capable of inducing cytokine independent cell growth [41–44, 46]. These programs are most useful in conjunction with other methods of predicting PTCL critical mutations, such as identification of recurrent mutations in known cancer pathways or genes mutated in other related cancers.

Publication of our data set will enable inclusion of the whole exome sequencing data from these primary PTCL patient samples in future research and analysis. Further assessments using this data, such as to determine copy number variation (CNV), may yield more insights into potential cancer drivers and therapeutic strategies. CNVs are important sources of genetic variation that involve significantly larger areas of the genome than SNVs and may lead to oncogenic phenotypes. Generally, CNV calling in exome data is more challenging and error prone than in whole genome sequencing and is still further complicated by complexity of tumor genomes. Recently developed tools to improve the accuracy of assessing CNVs in exome sequencing data, such as PatternCNV, may have utility in further identifying PTCL driver variations in the genomes of malignant T cells in this data set [64].

Our study, together with prior findings, expand the total number of PTCL samples analyzed for somatic mutations so that less common mutations identified in one study, such as γ_c_ K315E, may be exposed as recurrent, potentially critical mutations in the process of PTCL tumorigenesis and its relationship to normal T-cell biology to pave the way for the discovery and development of novel therapeutic targets.

## Supporting Information

S1 Fig Sanger sequencing chromatograms of γ_c_ signaling pathway transducers verify SNVs identified by whole exome sequencing. (TIF)

S2 Fig Sanger sequencing chromatograms of ATM verify SNVs identified by whole exome sequencing. (TIF)

S1 Table Descriptions of PTCL patient pathological samples. (TIF)

S2 Table Recurrent synonymous mutations identified in primary PTCL cases by subtype. (TIF)

S3 Table Somatic mutations identified in primary PTCL cases predicted to be oncogenic driver mutations by CHASM. (TIF)
