# Supplementary figures and images for: Concurrent Mutations in ATM and Genes Associated with Common γ Chain Signaling in Peripheral T Cell Lymphoma
Source: PLoS One. 2015 Nov 4;10(11):e0141906. doi: 10.1371/journal.pone.0141906 (PMC4633051; doi:10.1371/journal.pone.0141906)

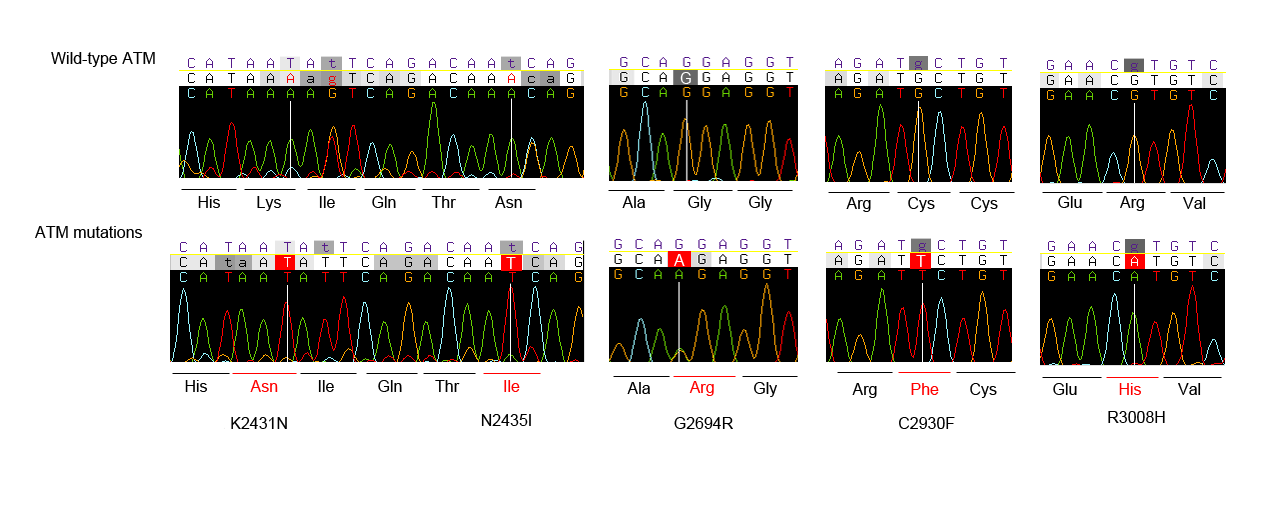

Supplement: S1 Fig — (TIF) [file pone.0141906.s001.tif]

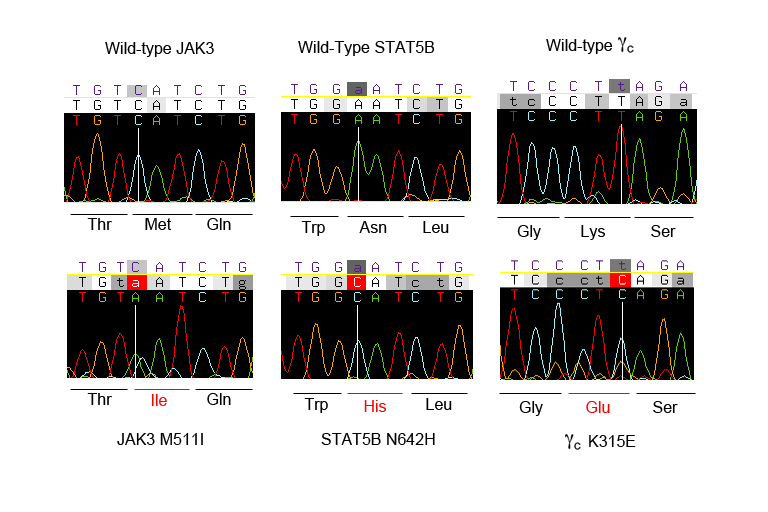

Supplement: S2 Fig — (TIF) [file pone.0141906.s002.tif]

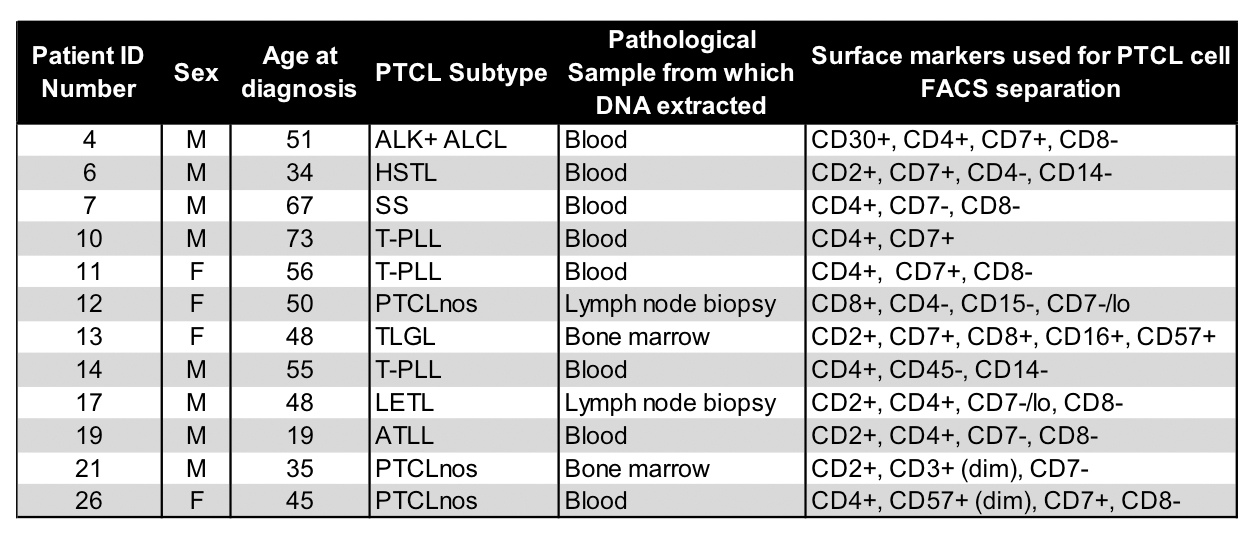

Supplement: S1 Table — (TIF) [file pone.0141906.s003.tif]

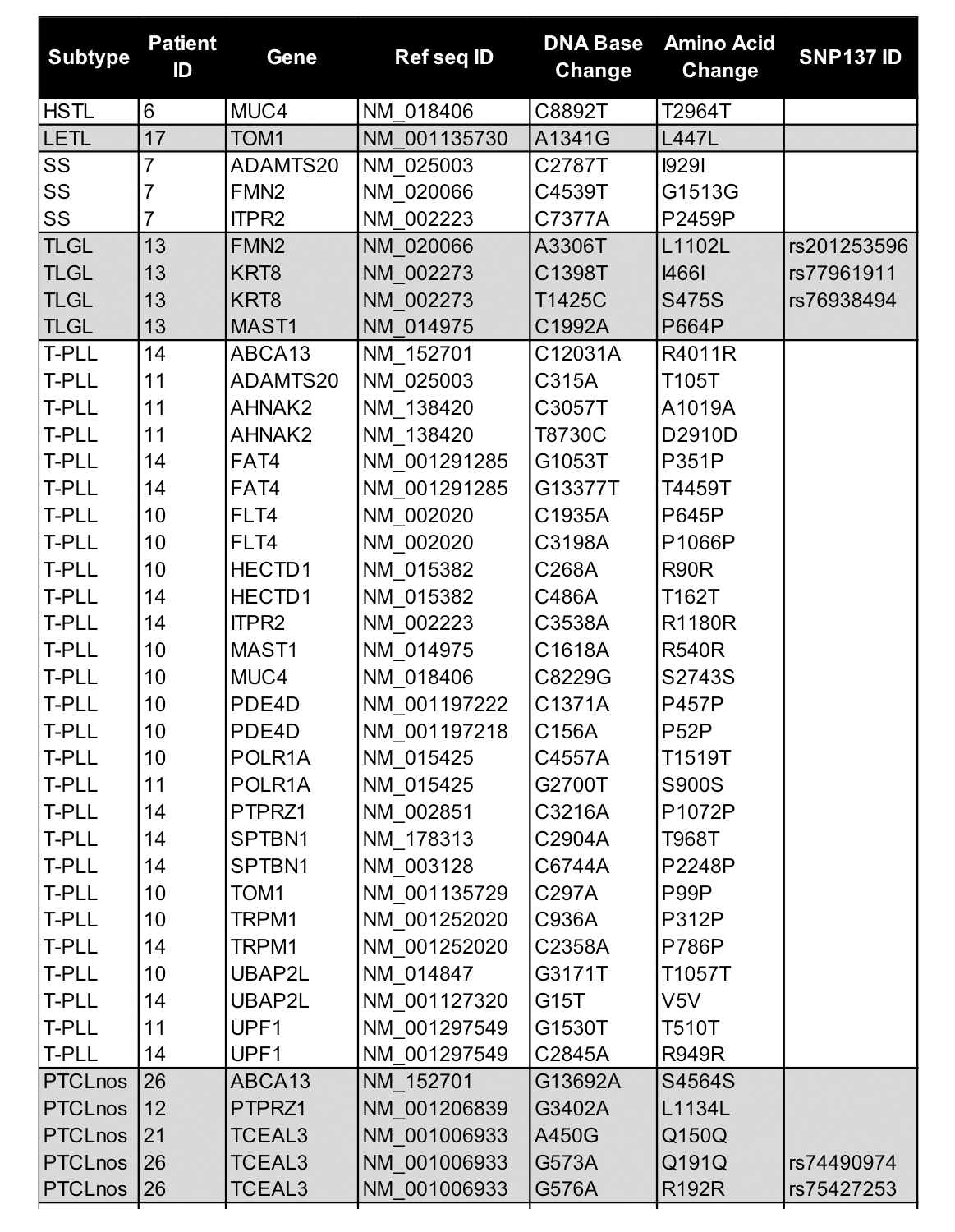

Supplement: S2 Table — (TIF) [file pone.0141906.s004.tif]

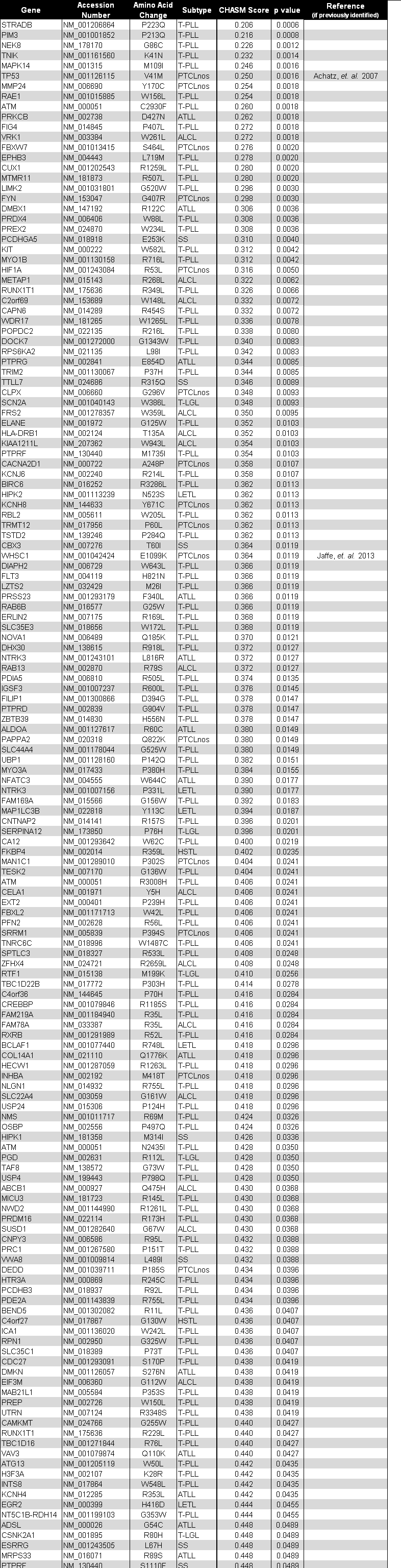

Supplement: S3 Table — (TIF) [file pone.0141906.s005.tif]
